# DRESS/DiHS syndrome induced by Propylthiouracil: a case report

**DOI:** 10.1186/s12902-023-01273-x

**Published:** 2023-01-23

**Authors:** Qiong Shen, Qingyao Wang, Huanhuan Zang, Ling Yu, Xiangguo Cong, Xinxin Chen, Lei Chen

**Affiliations:** Department of Endocrinology, Suzhou Municipal Hospital Affiliated to Nanjing Medical University, 26 Daoqian Road, Suzhou, 215000 People’s Republic of China

**Keywords:** Propylthiouracil, Drug reaction with eosinophilia and systemic symptoms (DRESS), Drug-induced hypersensitivity syndrome (DiHS)

## Abstract

**Background:**

Drug reaction with eosinophilia and systemic symptoms (DRESS), also known as Drug-induced hypersensitivity syndrome (DiHS), is a severe adverse drug reaction. Propylthiouracil, a member of thiouracils group, is widely used in medical treatment of hyperthyroidism. Propylthiouracil is associated with multiple adverse effects such as rash, agranulocytosis hepatitis and antineutrophil cytoplasmic antibody (ANCA)-associated vasculitis, but rarely triggers DRESS/DiHS syndrome. Here, we describe a severe case of propylthiouracil-induced DRESS/DiHS syndrome.

**Case presentation:**

A 38-year-old female was treated with methimazole for hyperthyroidism at first. 4 weeks later, the patient developed elevated liver transaminase so methimazole was stopped. After liver function improved in 2 weeks, medication was switched to propylthiouracil therapy. The patient subsequently developed nausea and rash followed by a high fever, acute toxic hepatitis and multiple organ dysfunction (liver, lung and heart), which lasted for 1 month after propylthiouracil was started. According to the diagnostic criteria, the patient was diagnosed of DRESS/DiHS syndrome which was induced by propylthiouracil. As a result, propylthiouracil was immediately withdrawn. And patient was then treated with adalimumab, systematic corticosteroids and plasmapheresis in sequence. Symptoms were finally resolved 4 weeks later.

**Conclusions:**

Propylthiouracil is a rare cause of the DRESS/DiHS syndrome, which typically consists of severe dermatitis and various degrees of internal organ involvement. We want to emphasize through this severe case that DRESS/DiHS syndrome should be promptly recognized to hasten recovery.

## Background

Hyperthyroidism is generally considered to be caused by the sustained overproduction and release of hormone by the thyroid itself. In most cases, the thyroid function test shows high level of free triiodothyronine (FT3) and/ or free thyroxine (FT4), and suppressed thyroid stimulating hormone (TSH). Antithyroid drugs, including propylthiouracil (PTU) and methimazole (MMI), were preferred by many clinicians and patients since they were convenient and non-invasive. PTU is a member of the thiouracil class of drugs, which reduces the synthesis of thyroid hormone by interfering with thyroid peroxidase (TPO)-mediated iodine oxidation and organicication. PTU has been widely used for patients with hyperthyroidism and it is also recommended that pregnant patients with hyperthyroidism should use PTU to prevent congenital malformation during the first three trimester [1]. Previous literatures have reported that PTU has side effects such as liver damage, agranulocytosis, urticaria and antineutrophil cytoplasmic antibody (ANCA)-associated vasculitis in clinic [2]. Drug reaction with eosinophilia and systemic symptoms (DRESS), also known as drug-induced hypersensitivity syndrome (DiHS), is one of the severe cutaneous adverse reaction syndromes, characterized by a heterogeneous group of clinical manifestations such as acute and extensive skin lesions, fever, lymphadenopathy, eosinophilia, multiple organ injuries like liver (increased alanine aminotransferase and aspartate aminotransferase, ALT and AST), lung, renal and cardiac system [3, 4]. According to previous reports, the causative drugs of DRESS syndrome include anticonvulsants, antimicrobial agents, antiviral agents, antipyretic agents, etc. [5]. In rare cases, PTU may lead to DRESS/DiHS. Until now, only 3 cases of PTU-induced DRESS/DiHS syndrome have been reported [6–8]. Here, we are reporting another severe case of a female who developed DRESS/DiHS syndrome after taking PTU for 4 weeks and conducted a literature review of DRESS/DiHS syndrome.

## Case presentation

A 38-year-old woman was presented to our hospital with symptoms of nausea and erythroderma for 10 days, and fever for 2 days. She was allergic to Sulfonamides drugs. Meanwhile, she denied any history of hypertension, diabetes, hepatitis or other malignant disease.

**Fig. 1:**
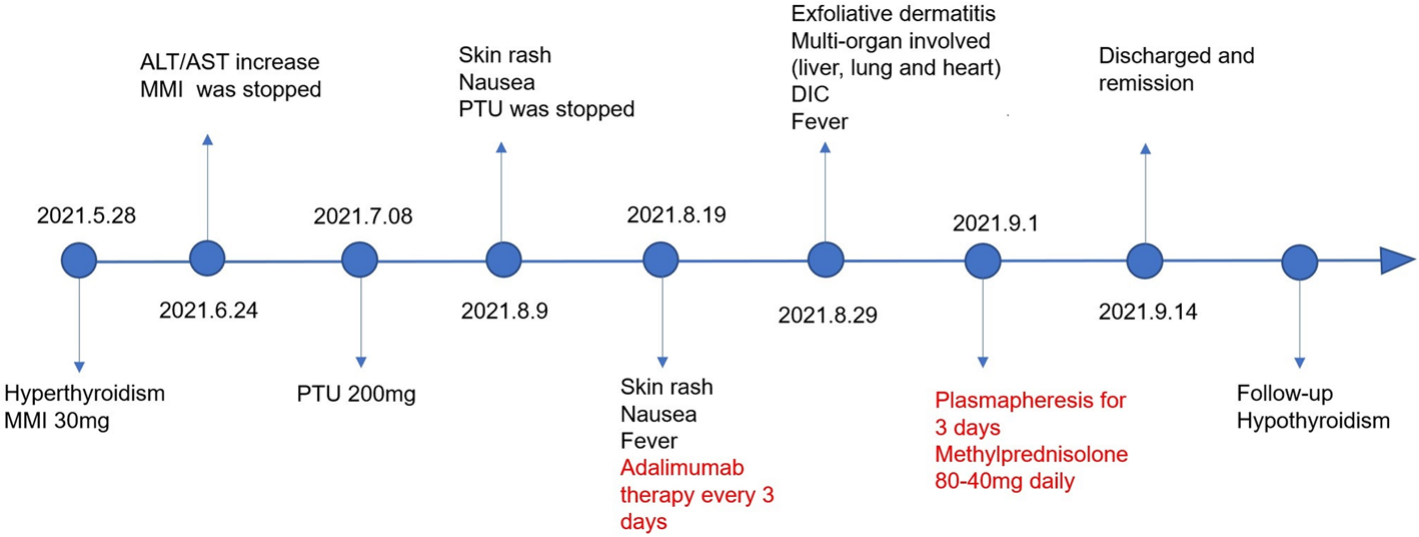
The diagnosis and treatment timeline of the case

She was prescribed with methimazole (30 mg/d) and propranolol (30 mg/d) for treatment of hyperthyroidism (FT3 10.26 pmol/L (2.43–6.01 pmol/L), FT4 34.22 pmol/L (9.01–19.05 pmol/L), TSH 0 μIU/ml (0.35–4.94 μIU/mL), positive TRAb 2.39 IU/L (0.00–1.75 IU/L)) in Outpatient Department on May 28, 2021. Methimazole was discontinued for the abnormal liver function (ALT 143 U/L, AST 60 U/L) on June 24. Propylthiouracil, 200 mg/d, was applied when her liver function returned to normal on July 8. However, she developed diffuse erythematous rash, which appeared on the cheeks, neck and upper limbs, and caused itching on August 9. Although propylthiouracil was discontinued immediately, erythema had spread to the chest and distal extremities, and she was admitted to our department 10 days later. The timeline for diagnosis and treatment was summarized in Fig. 1.

Physical examination findings were: high fever (39.1 °C), sinus tachycardia, diffuse erythema spread all over the trunk and extremities, submandibular lymphadenopathy and facial edema. Laboratory findings showed increased leukocytes (19.57 × 10ˆ9/L), absolute eosinophilia (1.19 × 10ˆ9/L), 10% atypical leukocytosis, abnormal liver function (ALT 635 U/L, AST 231 U/L), increased ALP (157 U/L). The results of the tests for hepatitis A, B, and C, Epstein-Barr virus (EBV) and Cytomegalovirus (CMV) DNA quantification were negative. The levels of C reactive protein (CRP) and prolactin were normal. Serological tests of ANCA and IgG4 were also negative.

## Treatment and prognosis

**Fig. 2:**
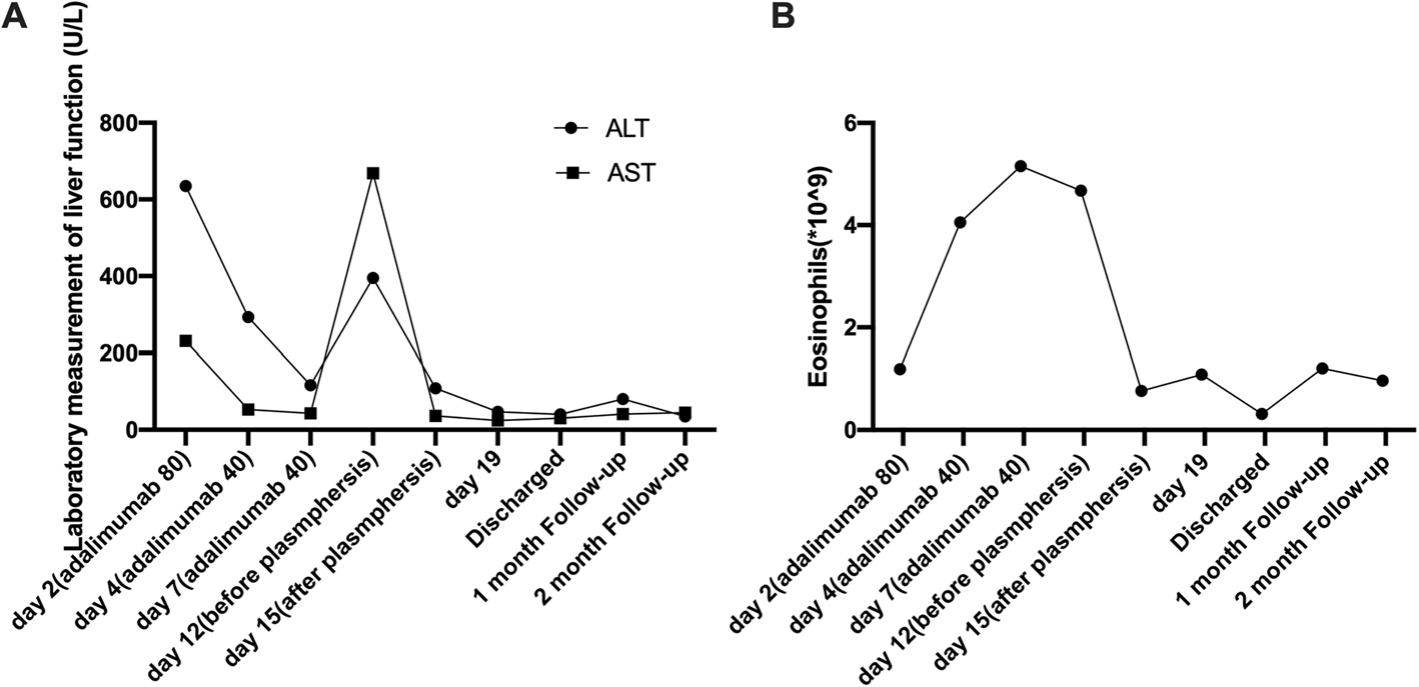
The detection of liver function and eosinophils throughout the course of disease. Liver function throughout the course of disease was shown in (**A**) and the absolute counts of eosinophils were shown in (**B**), respectively

With a tentative diagnosis of propylthiouracil-induced hypersensitivity syndrome, we started adalimumab injection. Adalimumab (tumor necrosis factor-α inhibitors; TNF-α inhibitors) was administrated via subcutaneous injection every 3 days with 80 mg (dose doubled) at first and 40 mg thereafter. Supportive therapy such as hydration and topical corticosteroids was also applied. After the first adalimumab injection, serum ALT and AST levels decreased significantly while WBC and eosinophils were continued to increase and reached the peak on Day 6 (Fig. 2). Bone marrow biopsy was then applied and showed 10% atypical cells. The skin rash gradually merged but itching worsened after the first injection of adalimumab, and then desquamation began 3 days later. Subsequently, she developed a persistent high fever, multiple serious membrane effusion, disseminated intravascular coagulation (DIC), multiple organ dysfunction (liver injury, pneumonia and heart failure), multiple oral ulcers. And rashes in the extremities and trunk parts started to exfoliate. The human EBV DNA, which was negative on admission, was detected by polymerase chain reaction during this period. She was subsequently transferred to intensive care unit in our hospital and treated comprehensively with a plasmapheresis for 3 days. Then methylprednisolone (80 mg/d) was administrated for 3 days, and then reduced to 40 mg/d. 4 weeks later, the patient’s symptoms disappeared, the levels of ALT and AST and eosinophil counts returned to normal.

Thyroid function changed considerably during the course of the treatment. On September 10, the patients showed normal levels of FT3 (3.49 pmol/L), high levels of FT4 (19.17 pmol/L) and decreased TSH (0.0415 μIU/L). Later the patient became mildly hypothyroidism (FT3 < 2.3 pmol/L, FT4 7.27 pmol/L, TSH 12.6322 μIU/ml) and began to take levothyroxine 75 μg/day on September 27.

**Fig. 3:**
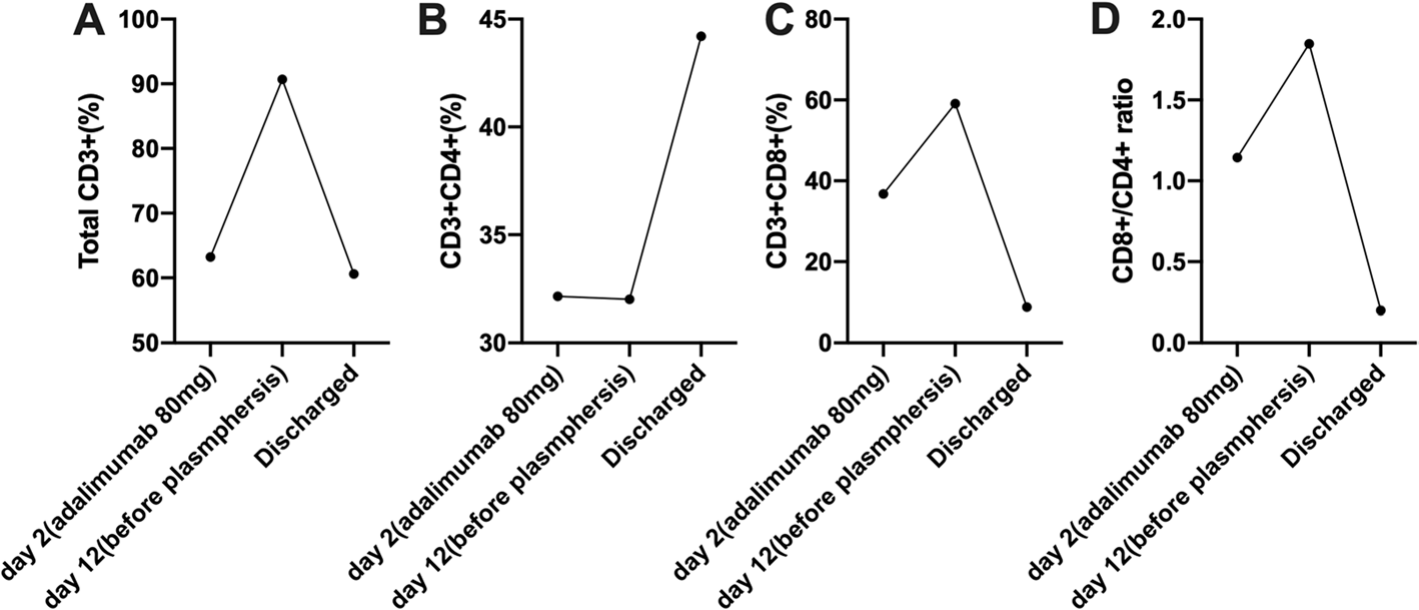
The detection of CD4 + T and CD8 + T cells in peripheral blood of the patient throughout the course of disease. The percentages of total CD3 + T cells (**A**), CD3 + CD4 + T cells (**B**), CD3 + CD8 + T cells (**C**) were detected in day 2, day 12 and discharge day, respectively. The ratio of CD8+/CD4 + T cells was shown in (**D). **Day 2 was the day after the first injection of adalimumab (80 mg), day 12 was the day before plasmapheresis

T lymphocytes were detected by flow cytometry on the Day 2, Day 12 (before plasmapheresis, exacerbation of syndrome) and discharge day, which was shown in Fig. 3, CD3 + T and CD3 + CD8 + T cells increased significantly with the exacerbation of the disease, but decreased to normal while in remission.

## Discussion and conclusions

Drug reaction with eosinophilia and systemic symptoms (DRESS) was first reported by Merrit and Puttnam in 1938 and termed by Bocquet et al. to distinguish this syndrome from other drug-induced cutaneous adverse reaction without eosinophilia [9, 10]. In 1988, the terminology of DiHS was proposed in Japan to emphasize the reactivation of HHV-6 in the majority of patients [11]. Ushigome et al. have demonstrated that DRESS syndrome and DiHS were actually the same disease [12]. DRESS syndrome could be diagnosed on scoring system by RegiSCAR group, and DiHS could be diagnosed on criteria by Japanese Research Committee on Severe Cutaneous Adverse Reaction (J-SCAR) group [13, 14]. According to the RegiSCAR criteria, our patient scored 8 points including fever (0), eosinophilia greater than 1.5*10 ˆ9/L (2 points), atypical lymphocytosis (1 points), enlarged lymph nodes (1 points), the area of the rash more than 50% (1 points), organ involvement including heart, lung and liver (2 points), excluding other causes (1 points), so the probability of DRESS was categorized as “Definite”. According to J-SCAR group criteria, the patient could be diagnosed of atypical DiHS by the presence of maculopapular rash developed > 3 weeks after starting with a limited number of drugs, prolonged clinical symptoms after discontinuation of the causative drug, fever (> 38 °C), liver abnormalities (ALT > 100 U/L), leukocyte abnormalities including leukocytosis (> 11 × 10^9/L), atypical lymphocytosis (> 5%) and eosinophilia (> 1.5 × 10^9/L), lymphadenopathy.

The first and most important treatment of DRESS/DiHS syndrome is to withdraw the inciting drugs immediately. The drug that causes the syndrome should be PTU in our case. A distinctive feature of DRESS/DiHS syndrome is a long latent period after taking culprits drugs. It usually takes 2–6 weeks to develop the symptoms and signs after exposure to a culprit medication. The patient took MMI for 4 weeks and no DRESS symptoms were induced. Maculopapular rash was developed 4 weeks after taking PTU and aggravated after PTU was stopped. This was consistent with the other distinctive features of syndrome, that is, the worsening of clinical symptoms after the causative drugs withdrawal. Therefore, based on the timing and features of the eruption and the drug intake history, PTU was the causative drug in this case. However, PTU is a rare cause of DRESS/DiHS. We performed a literature search and only 3 cases of PTU inducing DRESS syndrome were reported (Table 1) [6–8], including 3 females, aged 14–34. Among the cases of PTU-induced DRESS/DiHS reported in previous literatures, 2 cases were resolved with corticosteroids treatment and the other case received symptomatic treatment.

**Table 1:** Literatures review of propylthiouracil induced DRESS syndrome

Year	Author	Area	Age/Gender	Initial Symptoms	Organ involved	Treatment and Outcome	Preceding events	Regi SCAR score
1991	Ping-Ching Fong	Hong Kong	31/Female	polyarthritis, fever and bilateral deafness	hearing organ	ketoprofen and potassium iodine, remission	Not report	Data is insufficient
2006	Zehra Aycan	Turkey	14/Female	fever, cough, chest pain and leg swelling	heart, lung	methylprednisolone, remission	Not report	4 points at most
2010	Y-M Ye	Korea	34/Female	febrile, diffuse erythematous rash, facial edema, itching, cervical lymphadenopathy, and oral mucosal involvement	liver	prednisolone, remission	Thyroiditis	6 points

Systemic corticosteroids remained to be the first choice to treat DRESS/DiHS syndrome in many reviews [3, 4, 13–16]. The recommended initial dose of corticosteroids was 0.5–1.0 mg/kg/d. In some mild cases, adequate hydration or topical corticosteroids could also be effective [17]. There was still a lack of consensus in the treatment for DRESS/DiHS syndrome, since the syndrome and severity of the disease were heterogeneous in clinic. It was recommended that systematic corticosteroids treatment or IVIG should be considered in the presence of signs of severity (transaminase levels > 5 times normal, renal involvement, pneumonia, hemophagocytosis, and cardiac severity, etc) or life-threatening signs (ie, hemophagocytosis with bone marrow failure, encephalitis, severe hepatitis, renal failure, and respiratory failure), and topical corticosteroids treatment in the absence of all these signs [18]. But side effects caused by corticosteroids drug, such as liver damage, gastric ulcer, osteoporosis, impaired glucose tolerance, etc. should also be taken into consideration. Systemic corticosteroids therapy was related to the activation of the virus in DRESS syndrome patients in some reports [19].

Other therapies such as intravenous immunoglobulin, cyclosporine, cyclophosphamide, mycophenolate mofetil, rituximab and adalimumab were also reported to be effective in literatures [20–22]. Cyclosporine treatment was found to have an advantage in reduced disease progression and improved findings in clinical and laboratory markers compared with steroids treatment with a shorter length of hospital stay in a retrospective study conducted in 26 patients with DRESS (RegiSCAR score > 5) [23]. TNF-α inhibitors were also reported to be effective in a few cases of DRESS syndrome. Kim et al. reported a case of corticosteroid-induced DRESS that was successfully treated with a TNF-α inhibitor [24]. Leman et al. reported a DRESS case associated with lithium carbonate, which was successfully treated with a TNF-α inhibitor [25]. Our case was treated with adalimumab firstly and showed partial response (decreased liver function). Her skin rash and eosinophilia were not ameliorated by initial treatment with adalimumab, and her symptoms progressed with life-threatening multi-organ involvement after the third dose of adalimumab injection. Then, systemic corticosteroids and plasmapheresis were applied. Due to the heterogeneity of the syndrome and sporadic case reports of immunotherapy for DRESS/DiHS syndrome, it is necessary to design rigorous large-sample RCT studies in the future to verify which immunotherapy is safe and effective.

The pathophysiological mechanisms of DRESS/DiHS syndrome were not well understood. Genetic, viral reactivation and immune interaction were involved in the pathogenesis of the disease [26]. Various Studies showed that the risk of hypersensitivity syndrome induced by different drugs is significantly related with HLA alleles [27, 28]. To date, the relationship between HLA alleles and PTU-induced DRESS/DiHS syndrome remains unclear. By the detection of lymphocyte subsets (Fig.3), we found that CD3 + T and CD3 + CD8 + T cells increased significantly with the exacerbation of disease, which is consistent with DRESS syndrome that is a delayed type IV hypersensitivity of drug reaction [29]. Jun Niu et al. have pointed out that it is the extent of fluctuation of CD8 + T cell clones,not that of CD4 + counterparts that is correlated positively with the severity of clinical manifestations and virus-mediated pathogenic CD8 + T cell clones may be a critical key leading to the pathogenicity of DRESS syndrome [30]. In addition, EBV-driven CD8 + T cells can cause cross-reactive damage to multiple organs and a high viral load and antibody titers are closely linked with bad outcomes [31].

The imbalanced and considerably increased T cells in acute phase may also be responsible for several autoimmune diseases after DRESS/DiHS remission. Several autoimmune conditions, including autoimmune thyroiditis, systemic lupus erythematosus, type 1 diabetes mellitus, and autoimmune hemolytic anemia, may occur after DRESS/DiHS syndrome [26, 32]. According to the previous reports, thyroid diseases, including Graves’ disease, Hashimoto’s thyroiditis, and painless thyroiditis, are the most frequent complications in DRESS patients [33]. In our case, the patient was presented TRAb positive hyperthyroidism at first and needed antithyroid drugs therapy. After DRESS/DiHS, she was presented with hypothyroidism with negative TRAb (on September 10)and high titers of thyroid peroxidase antibodies (TPOAb) and anti-thyroglobulin (TgAb) which was more likely to be Hashimoto’s thyroiditis (on September 27). Of note, TPOAb and TgAb increased gradually during the treatment at admission. She needed supplemental therapy of levothyroxine since her thyroid function test showed hypothyroidism in the outpatient department. The underlying mechanisms of this phenomenon was possibly related to the increased CD8 + T cells in DRESS/DiHS syndrome since CD8 + T cells were also involved in the production of TPOAb and TgAb [34].

In summary, PTU is a rare cause of the DRESS/DiHS syndrome, which typically consists of severe dermatitis and various degrees of internal organ involvement. We want to emphasize with this severe case that DRESS/DiHS syndrome should be promptly recognized during the early period of treatment to hasten recovery.

## Abbreviations

DRESS: Drug reaction with eosinophilia and systemic symptoms
DiHS: Drug-induced hypersensitivity syndrome
FT3: free triiodothyronine
FT4: free thyroxine
TSH: thyroid stimulating hormone
PTU: propylthiouracil
MMI: methimazole
TPO: thyroid peroxidase
ALT: alanine aminotransferase
AST: aspartate aminotransferase
EBV: Epstein-Barr virus (EBV)
CMV: Cytomegalovirus
CRP: C reactive protein
TNF-α: tumor necrosis factor –α
DIC: disseminated intravascular coagulation
TPOAb: thyroid peroxidase antibodies
TgAb: anti-thyroglobulin.
